# Optically accessible, 3D-printed flow chamber with integrated sensors for the monitoring of oral multispecies biofilm growth *in vitro*

**DOI:** 10.3389/fbioe.2024.1483200

**Published:** 2024-11-11

**Authors:** Nicolas Debener, Nils Heine, Beate Legutko, Berend Denkena, Vannila Prasanthan, Katharina Frings, Maria Leilani Torres-Mapa, Alexander Heisterkamp, Meike Stiesch, Katharina Doll-Nikutta, Janina Bahnemann

**Affiliations:** ^1^ Institute of Technical Chemistry, Leibniz University Hannover, Hannover, Germany; ^2^ Department of Prosthetic Dentistry and Biomedical Materials Science, Hannover Medical School, Hannover, Germany; ^3^ Lower Saxony Center for Biomedical Engineering, Implant Research and Development (NIFE), Hannover, Germany; ^4^ Institute of Production Engineering and Machine Tools, Leibniz University Hannover, Hannover, Germany; ^5^ Institute of Quantum Optics, Leibniz University Hannover, Hannover, Germany; ^6^ Institute of Physics, University of Augsburg, Augsburg, Germany; ^7^ Centre for Advanced Analytics and Predictive Sciences (CAAPS), University of Augsburg, Augsburg, Germany

**Keywords:** 3D printing, oral biofilm, *in vitro* model, microfluidic flow chamber, infection, dysbiosis

## Abstract

The formation of pathogenic multispecies biofilms in the human oral cavity can lead to implant-associated infections, which may ultimately result in implant failure. These infections are neither easily detected nor readily treated. Due to high complexity of oral biofilms, detailed mechanisms of the bacterial dysbiotic shift are not yet even fully understood. In order to study oral biofilms in more detail and develop prevention strategies to fight implant-associated infections, *in vitro* biofilm models are sorely needed. In this study, we adapted an *in vitro* biofilm flow chamber model to include miniaturized transparent 3D-printed flow chambers with integrated optical pH sensors – thereby enabling the microscopic evaluation of biofilm growth as well as the monitoring of acidification in close proximity. Two different 3D printing materials were initially characterized with respect to their biocompatibility and surface topography. The functionality of the optically accessible miniaturized flow chambers was then tested using five-species biofilms (featuring the species *Streptococcus oralis*, *Veillonella dispar*, *Actinomyces naeslundii*, *Fusobacterium nucleatum*, and *Porphyromonas gingivalis*) and compared to biofilm growth on titanium specimens in the established flow chamber model. As confirmed by live/dead staining and fluorescence *in situ* hybridization via confocal laser scanning microscopy, the flow chamber setup proved to be suitable for growing reproducible oral biofilms under flow conditions while continuously monitoring biofilm pH. Therefore, the system is suitable for future research use with respect to biofilm dysbiosis and also has great potential for further parallelization and adaptation to achieve higher throughput as well as include additional optical sensors or sample materials.

## 1 Introduction

Implant-associated infections that can lead to tissue destruction around medical implants present a significant challenge to modern dentistry. The term peri-implant mucositis describes reversible inflammatory reactions in the soft tissues surrounding a dental implant (Zitzmann and Berglundh, 2008; Heitz Mayfield and Salvi, 2018). This can develop into irreversible peri-implantitis, which is associated with the loss of supporting bone (Jepsen et al., 2015). Indeed, 26% of patients with a dental implant developed peri-implantitis after 5 years, which may ultimately result in implant failure (Dreyer et al., 2018).

Implant-associated diseases are caused by complex pathogenic bacterial biofilms that form on surfaces in the oral cavity. Biofilms are communities of one or more microorganisms that adhere to surfaces and/or to each other, which are incorporated into a self-produced extracellular biopolymer matrix (Costerton et al., 1995; Flemming and Wingender, 2010; Høiby et al., 2015). Early colonizers of oral bacterial biofilms – such as gram-positive *Streptococci* and *Actinomyces* – can adhere to saliva pellicles adsorbed on the implant surface and form initial commensal biofilms (Kolenbrander et al., 2010), to which other oral bacteria then tend to co-adhere. While early biofilms are associated with a healthy host tissue (Roberts and Darveau, 2002), changes in the biofilm network can lead to the development of pathogenic biofilms (Zhang et al., 2022) which de-regulate the host’s immune response, leading to inflammatory reactions (Wang, 2015). One factor leading to peri-implantitis is proposed to be a shift in the species distribution towards anaerobic gram-negative bacteria. For example, a number of studies have demonstrated a higher abundance of *Porphyromonas* and *Fusobacterium nucleatum* in peri-implantitis sites in comparison to that seen in healthy implant sites (Koyanagi et al., 2010; Apatzidou et al., 2017; Sanz‐Martin et al., 2017; Al-Ahmad et al., 2018).

Biofilms are relatively difficult to treat due to their inherent resilience towards antibiotics and the host immune response. Bacteria in biofilms exhibit reduced metabolic activity, are protected by the extracellular matrix in which they thrive, and have easier access to plasmid transfer (Donlan and Costerton, 2002; Fu et al., 2021). This leads to a 10-to-1000-fold higher resistance to antimicrobials when compared to planktonic bacteria (Davies, 2003). Furthermore, due to missing and reduced vascularization in implants and surrounding tissue, respectively, this resilience displayed towards systemic therapies is actually further enhanced for implant-associated biofilms (Smeets et al., 2014). A deeper understanding of oral biofilms together with a controlled reproduction of their formation are essential to enable the early detection of on setting implant-associated infections and to establish new therapeutic strategies.

To study biofilm-related research questions within a simplified system, numerous *in vitro* biofilm models have been developed that differ from one another with respect to both the bacterial species and their interactions within the biofilm (Blanc et al., 2014). The commercial Calgary biofilm device consists of a lid with pegs that fit onto 96-well plates – enabling the cultivation of biofilms under static conditions (Ceri et al., 1999). This device can be utilized for high-throughput testing of the effect of antimicrobial substances on dental biofilms or for the assessment of antimicrobial coatings (Soares et al., 2015; Ghezzi et al., 2023). Given that biofilm characteristics are among other factors dependent on the hydrodynamic environment (Purevdorj et al., 2002), however, flow biofilm models were developed to more closely resemble the situation *in vivo*. Corbin et al. (2011) used a flow cell model including a peristaltic pump to investigate the impact of mouthrinse actives on dental biofilms. The impact of shear stress on biofilm characteristics, including biofilm thickness and surface coverage, has been demonstrated in a variety of custom-made flow chamber setups, which were operated under conditions of varying flow rates (Recupido et al., 2020; Tsagkari et al., 2022). As flow chamber-based biofilm models are more complex than static systems, they are typically designed for a specific readout. Consequently, their adaptability and parallelizability are limited. In an attempt to combine the benefit of high throughput of static biofilm models with the physiological conditions in flow cell models, microfluidic models such as the BioFlux can also be used (Benoit et al., 2010; Nance et al., 2013; Díez-Aguilar et al., 2018).

In order to study the growth of oral bacterial biofilms on implant materials, Rath et al. (2017) developed a flow chamber system with integrated titanium specimen. It allows for the cultivation of multispecies biofilms under controlled flow conditions as well as subsequent microscopic analysis of the grown biofilms through a glass slide. The flow chamber system was subsequently modified from a closed circuit to an open system, and its functionality was validated by growing four-species biofilms consisting of *Streptococcus oralis*, *Actinomyces naeslundii*, *Veillonella dispar* and *Porphyromonas gingivalis –* resulting in the “Hannoverian oral biofilm implant flow chamber (HOBIC) model” (Kommerein et al., 2018). However, this model does not allow for a continuous monitoring of biofilm development or metabolic processes due to the limited optical access.

With additive manufacturing, the creation of custom systems has increasingly become possible even within the constraints of relatively limited financial means. Computer-aided design (CAD) programs can be used to fabricate three-dimensional complex structures layer by layer with the assistance of 3D printers – and thanks to the plethora of 3D printing techniques that are now available, a wide variety of materials (e.g., ceramics, polymers, acrylate resins, etc.) can be deployed for these ends (Gross et al., 2014). Kristensen et al. (2020) presented a 3D-printed, disposable flow cell that is suitable for microscopic analysis. This chamber permitted the investigation of the impact of flow conditions on dental biofilms, while maintaining material costs below 1 $. To combine optical accessibility with electrochemical impedance spectroscopy (EIS) sensors, McGlennen et al. (2023) published a 3D-printed flow cell that can be mounted to a platform to allow for confocal laser scanning microscopy (CLSM) analysis during biofilm growth. Therefore, 3D printing appears to be an excellent tool to produce optically accessible flow chambers for the cultivation of oral biofilms, which also enables the creation of systems that can be rapidly adjusted to accommodate different sample materials or allow for multiple subsequent analyses.

**FIGURE 1:**
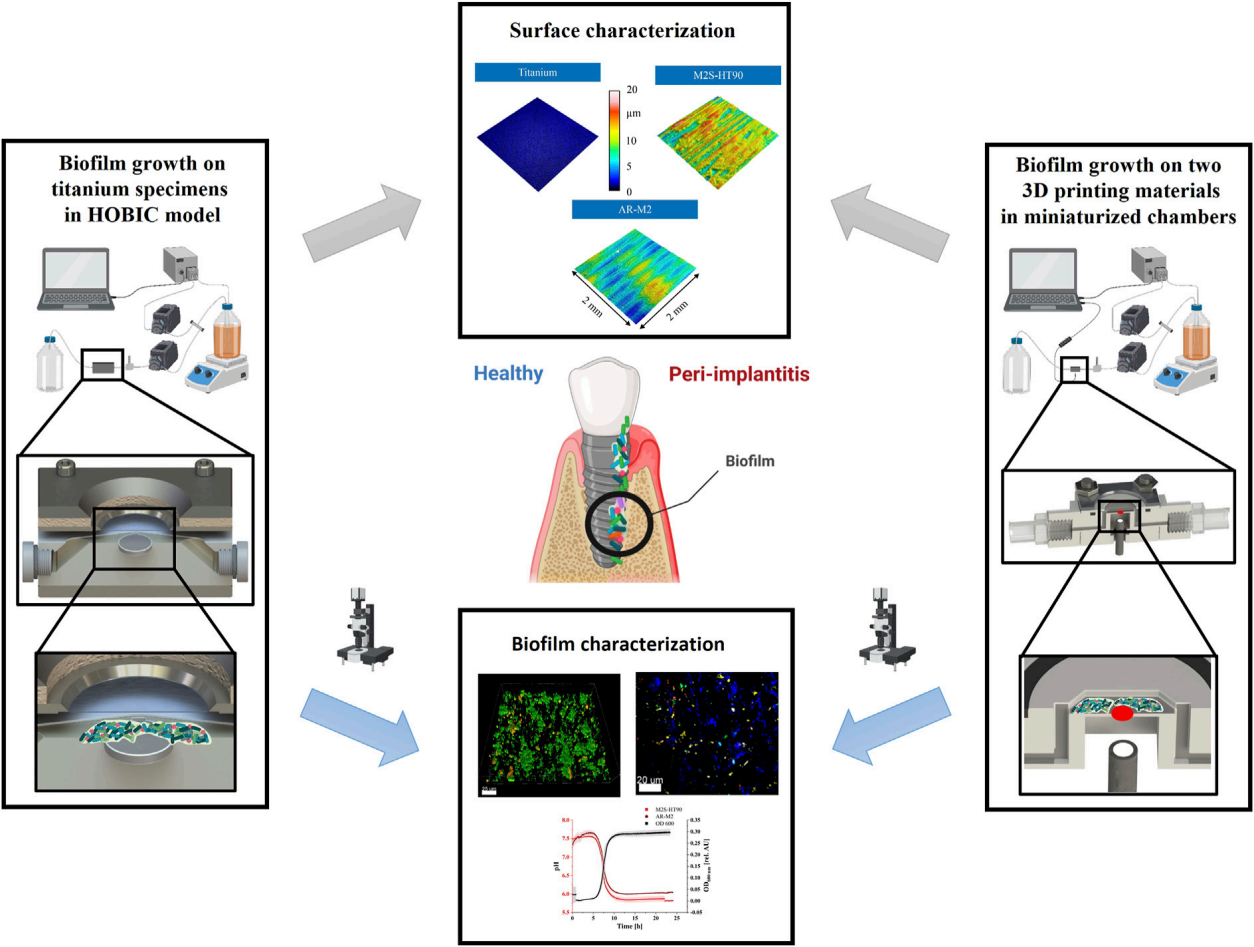
Schematic representation of the flow chamber setups and analyses (parts of this figure were created using biorender.com).

The aim of this study was to develop a microfluidic flow chamber that allows for the direct examination of biofilm growth. For this purpose, the flow chambers of the HOBIC model introduced by Kommerein et al. (2018) were replaced by miniaturized chambers fabricated by two different high-resolution MultiJet 3D printers with integrated pH-sensitive sensor spots to monitor the pH value inside the main channel in close proximity to the growing biofilms. The printing materials were characterized by confocal microscopy and water contact angle measurements and assessed for any possible cytotoxic effects of leachables hindering the growth of bacteria. Bacterial oral biofilms consisting of the five species *S. oralis*, *A. naeslundii*, *V. dispar*, *F. nucleatum* and *P. gingivalis* were grown in the 3D-printed devices for 24 h and analyzed by confocal laser scanning microscopy and fluorescent *in situ* hybridization (FISH) to quantify both biofilm volume and live/dead distribution, as well as to qualitatively analyze the species’ distribution. A graphical illustration of the experimental approach is presented in Figure 1. This work demonstrates the compatibility of 3D-printed microfluidic chambers for analysis of complex multispecies biofilms and shows their prospects for high-throughput applications with online monitoring of biofilms’ physiological properties.

## 2 Materials and methods

### 2.1 Fabrication of microfluidic flow chambers

The 3D-printed components of the presented flow chamber setup were designed using SolidWorks 2020 software (Dassault Systems Deutschland GmbH, Stuttgart, Germany) and fabricated using two different high-resolution 3D printers (ProJet^®^ MJP 2500 plus, 3D Systems, Rock Hill, SC, United States and AGILISTA 3,200 W, Keyence Deutschland GmbH, Neu-Isenburg, Germany). For the former 3D printer, the printing material VisiJet^®^ M2S-HT90 (3D Systems) was utilized as building material, while VisiJet^®^ M2 Sup (3D Systems) served as supporting material. Following printing, the parts were subjected to a post-processing procedure as described by Winkler et al. (2022). In short, the components were subjected to low temperatures, removed from the printing platform, and underwent repeated cleansing with water and detergent in a heat steam and ultrasonic bath. For the latter 3D printer, AR-M2 and AR-S1 (Keyence Deutschland GmbH) were used as building and supporting material, respectively, and post-processed as previously reported by Siller et al. (2020). In brief, the components were transferred to an ultrasonic water bath and cleaned with and without the use of detergents. Following these post-processing steps, the parts were incubated for 1 h at 37°C in 70% (v/v) ethanol and then washed with distilled water.

In order to enhance the stability of the lid as well as to uniformly distribute the screws’ pressure, aluminum frames were cut out of leftover pieces. To facilitate subsequent microscopic analyses, holes with a diameter of 13 mm were drilled in the center of the frames.

### 2.2 Assembly and sterilization of the microfluidic flow chambers

To ensure planar surfaces, the 3D-printed lids were placed in a drying oven with weights at 70°C for 1 h. Then, a glass panel was glued in a recess in the lid using a thin layer of epoxy adhesive Loctite^®^ EA-M-31CL™ (Henkel AG & CO.KGaA, Düsseldorf-Holthausen, Germany), which is biocompatible according to manufacturer’s specification (Henkel AG & Co.KGaA, 2020). Weights were added and the adhesive was left to cure overnight at room temperature, prior to sterilization under UV light.

Self-adhesive pH sensor spots SP-LG1-SA (PreSens Precision Sensing GmbH, Regensburg, Germany) were cut to a diameter of 2 mm and glued to the center of the main flow chamber. The underside of the chamber was first polished with 70% (v/v) isopropanol and then subsequently re-polished with a dry cloth in order to increase transparency. All 3D-printed components (with the exception of the lid), sealing rings, aluminum frames, and Luer adapters were heat-steam sterilized and then assembled and tightened with M3x12 set screws. Additionally, the Luer adapters were sealed with adhesive to prevent leaks due to heat deformation of the material. The pH sensor spots were recalibrated with PBS solutions with a total ionic strength of 130 mmol/L at six different pH values (7.5, 7.0, 6.5, 6.0, 5.5, and 5.0) at 37°C.

### 2.3 Surface characterization of 3D-printed material

To characterize the 3D-printed surfaces, plates with a diameter of 6 mm and a height of 2 mm were first fabricated with both 3D printing materials and then post-processed as described above.

Static water contact angles were measured at 22°C with the OCA 15 EC measuring device and SCA 20 software (Data Physics Instruments GmbH, Filderstadt, Germany) for the 3D-printed plates and titanium disks. Prior to the application of 2 µL water drops onto the samples, they were washed with isopropanol, dried with nitrogen, and grounded to avoid static charge. Images were captured 10 s after the drop was applied, and water contact angles were determined as the mean of the left and right water contact angles of 8 specimens of each 3D printing material, with three separate droplets per specimen (total n = 24). Statistical analysis was performed using a one-way ANOVA with Tukey’s test for multiple comparisons and α = 0.05.

Additionally, the surface topography of the two 3D printing materials and titanium control specimen (titanium grade 4) was examined. Due to the transparency of the printed samples, these were sputtered laterally with gold for 270 s using a Cressington Sputter Coater 108 auto (TESCAN GmbH, Dortmund, Germany) in a vacuum flushed with argon. The surface topography of the samples was measured at 20-fold magnification with an optical measuring device (DuoVario, Confovis GmbH, Jena, Germany). A measuring field of 2.3 mm × 2.3 mm was selected for the evaluation of surface topography using the software µsoft analysis premium 8.2 (Digital Surf, Besancon, France). The roughness parameters Rz, R_max_ and Ra were determined for 11 samples per material using a cut-off wavelength λ_c_ = 0.8 mm. Statistically significant differences were determined using a one-way ANOVA with Tukey’s test for multiple comparisons and α = 0.05.

### 2.4 Bacterial biocompatibility of printing material

Bacterial biocompatibility testing was performed according to ISO 10993-12 (International Organization for Standardization, 2021). Cubes with an edge length of 5 mm were produced with both 3D printing materials and post-processed as described above, before heat-steam sterilization. The extraction medium was prepared as previously described (Winkler et al., 2022). In short, the 3D-printed cubes were incubated in brain heart infusion medium (BHI, Oxoid Deutschland GmbH, Wesel, Germany) supplemented with 10 mg/L vitamin K (BHI + VitK, Carl Roth GmbH + Co. KG, Karlsruhe, Germany) at 37°C for 72 h.

*V. dispar* DSM 20735 (German Collection of Microorganisms and Cell Cultures GmbH, DSMZ, Braunschweig, Germany), *F. nucleatum* DSM 15643 (DSMZ), and *P. gingivalis* DSM 20709 (DSMZ) were anaerobically cultivated on fastidious anaerobe agar (Lab M Ltd., Heywood, United Kingdom) plates supplemented with 5% defibrinated sheep blood (Thermo Fisher Scientific Inc., Waltham, MA, United States) for 72 h. Afterwards, colonies were picked, transferred to BHI + VitK, and incubated at 37°C under anaerobic conditions for 18 h. Additionally, *S. oralis* ATCC^®^ 9811™ (American Type Culture Collection, ATCC, Manassas, VA, United States) and *A. naeslundii* DSM 43013 (DSMZ) were anaerobically cultivated for 18 h in BHI + VitK at 37°C. Anaerobic culture conditions were achieved using AnaeroGen™ bags (Thermo Fisher Scientific Inc.). The precultures were then centrifuged at 4,000 × g for 15 min, resuspended in fresh BHI + VitK and adjusted to an optical density at 600 nm (OD_600_) of 0.5. Biocompatibility tests were performed with suspensions of *S. oralis* as the main contributor to the biofilm, as well as with a mixture of the five bacterial species. For this purpose, equal volumes of the prepared bacterial suspensions were combined. Within a 96-well microtiter plate (Greiner Bio-One GmbH, Frickenhausen, Germany), 10 µL of the respective bacterial suspension were subsequently mixed with 90 µL of the extraction medium per well, resulting in an OD_600_ of 0.05. The plates were then anaerobically incubated at 37°C, and the OD_600_ was measured repeatedly using a microplate reader (M200 PRO, Tecan Group Ltd., Männedorf, Switzerland) to monitor bacterial growth. 100 μL of bacterial solution and 100 µL of extraction medium per well served as positive and negative control, respectively. Plain BHI + VitK was used as negative control for bacterial growth. Three independent precultures of *S. oralis* were prepared and each analyzed in triplicate over a 24-h period, resulting in a total of n = 9 wells per extraction medium and positive control. For evaluation, the mean optical densities of the respective negative controls were subtracted from the mean optical densities of the samples, to eliminate any potential influences on the turbidity caused by leachables. Statistical analysis was performed using a mixed-effects analysis with Tukey’s test for multiple comparisons for *S. oralis* and a two-way ANOVA with Tukey’s test for multiple comparisons for the five species mix, both with α = 0.05.

### 2.5 Biofilm formation within the microfluidic flow chambers

In previous studies, Kommerein et al. (2018) and Rath et al. (2017) have described a flow chamber system comprising of polyaryletherketone main flow chamber bodies and integrated titanium disks for the analysis of four-species biofilms. In brief, this system consists of a culture flask (containing sterile medium that is inoculated with the desired bacteria), an inline photometer as bypass to measure the optical density in the culture flask, a peristaltic pump that pumps the medium through the flow chambers at a specified flow rate, bubble traps to eliminate air bubbles in the tubes, the flow chambers themselves, and a waste bottle. The same system was employed in this work, but we replaced the previous flow chambers with the miniaturized 3D-printed units containing a pH-sensitive sensor spot within the main channel (Figure 1).

*S. oralis*, *A. naeslundii*, *V. dispar*, *F. nucleatum*, and *P. gingivalis* were cultivated as described above. Precultures were adjusted to an OD_600_ of 0.05, and 2.1 mL of each species were injected into the culture flask containing 1.5 L BHI + VitK. Due to the decreased size of the flow chamber compared to previous works, bacteria were guided through the system with an adjusted flow rate of 25 μL/min in order to match previous flow velocities. Biofilms were grown for 24 h at 37°C, while continuously measuring the OD_600_ in the culture flask as well as the pH value in the main channel of the flow chamber via a fiber optic based pH meter (pH-1 SMA LG1, PreSens Precision Sensing GmbH). The 24-h biofilm experiments were carried out as three separate biological replicates. Each replicate consisted of five chambers for both printing materials fed with bacteria from the same culture flask. As a reference, five-species 24-h biofilm experiments were conducted on the titanium control specimens (as described above) in the flow chamber system as described by Kommerein et al. (2018). For FISH staining, the flow chambers were integrated into the system in an inverted position to facilitate the formation of biofilms on the glass panel of the cover. This was due to the printing material interfering with the FISH probes and fluorescence microscopy. To determine statistically significant differences of pH, an unpaired t-test was performed with α = 0.05.

### 2.6 Biofilm analysis by fluorescence microscopy

After 24 h, the flow chambers were separated from the system, and biofilms were analyzed in the main channel on the 3D printing material around the sensor spots. To remove unbound bacteria, biofilms were washed for 20 min with phosphate buffered saline (PBS, Sigma Aldrich, St. Louis, MO, United States) at a flow rate of 100 μL/min.

With the same flow rate, a 1:2,000 dilution of the two fluorescent dyes of the Live/Dead BacLight Bacterial Viability Kit (Life Technologies, Darmstadt, Germany) in PBS were pumped through the system. Syto9 can penetrate intact bacterial membranes, while propidium iodide (PI) can only enter cells with damaged membranes. The dyes intercalate with the DNA and emit fluorescence signals at their respective emission wavelengths. After staining, biofilms were fixed with 2.5% (v/v) glutardialdehyde (Carl Roth GmbH + Co. KG) at 4°C for at least 15 min. The glutardialdehyde was pumped into the chambers at a flow rate of 100 μL/min, after which the flow chambers were removed from the system and stored at 4°C. Biofilms were then analyzed via confocal laser scanning microscopy (Leica TCS SP8, Leica Microsystems, Mannheim, Germany). Lasers with wavelengths 488 nm and 552 nm were used to excite Syto9 and PI, respectively. For Syto9, the detector was set to the wavelength range of 500–540 nm. The emission of PI was detected at the wavelength range of 675–750 nm. For each sample, image stacks with an x-y-size of 184.52 µm × 184.52 µm and a z-step-size of 2 μm at five random positions around the sensor spots were taken with a 63-fold magnification microscope objective (NA = 0.9). 3D biofilm reconstructions as well as live/dead distribution and biofilm volume calculations were performed using Imaris x64 8.4 software (Bitplane AG, Zurich, Switzerland). Biofilm areas that were stained with Syto9 are considered to consist of viable bacteria, while regions stained by propidium iodide were considered dead. Areas in which both dyes were found to be present were subtracted from the viable biofilm volume, since the membranes in these regions were damaged. Statistical analysis was performed using a Kruskal-Wallis test with Dunn’s test for multiple comparisons and α = 0.05.

To prepare the flow chambers for FISH analysis, 50% (v/v) ethanol was pumped through the system over the washed biofilms for 50 min with a flow rate of 100 μL/min. With the ethanol inside, the chambers were then removed from the system and stored at 4°C overnight to fixate the bacteria. The chambers were subsequently opened and left to dry under sterile conditions. FISH staining and CLSM analysis were performed as reported by Kommerein et al. (2018), the 16S rRNA probes used are listed in Supplementary Table S1 of the supporting information. Briefly, membranes were disrupted by treatment with 1 g/L lysozyme (Merck KGaA, Darmstadt, Germany) at 37°C for 10 min. After washing with pure ethanol, staining was done at 46°C for 30 min in a hybridization buffer containing the six fluorescently labeled probes. *F. nucleatum* was stained by two probes, sharing the same nucleotide sequence but with different dyes – resulting in co-localized blue and red fluorescence. After several washing steps, the samples were analyzed by CLSM taking image stacks with an x-y-size of 184.52 µm × 184.52 µm and a 2 µm z-step-size with a 63-fold magnification microscope objective (NA = 0.9). CLSM was performed using two sequences for each frame. In the first sequence, a 405 nm and a 552 nm laser were used and detectors were set to wavelength range 413–477 nm and 576–648 nm for blue and yellow, respectively. For the second sequence, a 488 nm and a 638 nm laser excited the samples and emission was recorded with detectors set to wavelength range 509–576 nm and 648–777 nm for green and red, respectively.

### 2.7 Statistical analysis

Data presentation and statistical analysis was done using the GraphPad Prism 8.4 (Graphpad Software Inc., San Diego, CA, United States) software as stated for the respective experiments.

## 3 Results

### 3.1 Design of the microfluidic flow chambers

**FIGURE 2:**
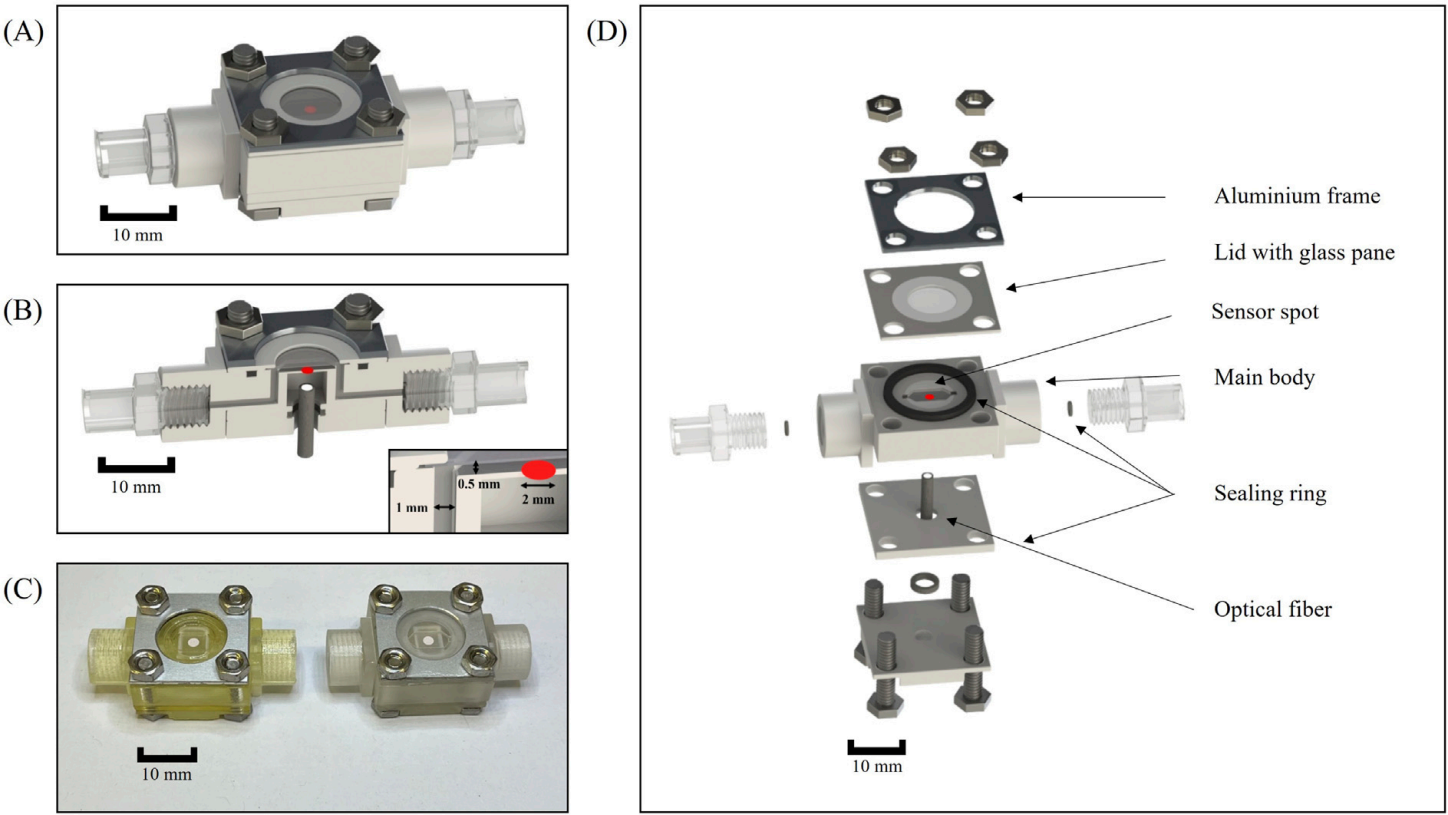
Microfluidic flow chamber setup. **(A)** CAD illustration of the fully assembled flow chamber. **(B)** Sectional view of the assembled flow chamber. **(C)** Photograph of the assembled flow chambers without Luer connectors. Left: Flow chamber fabricated with AGILISTA 3200 W using AR-M2 printing material. Right: Flow chamber manufactured with Projet MJP 2500 plus using the M2S-HT90 3D printing material. **(D)** Explosion view of the flow chamber with all additional parts.

The design of the flow chamber with all 3D-printed and additional parts is illustrated in Figures 2A–D. These chambers were integrated into the HOBIC model proposed by Kommerein et al. (2018). They replaced the previous flow chambers, which were shown in detail in Rath et al. (2017). The new microfluidic chambers consist of a 3D-printed body featuring an open top main channel with a pH-sensitive sensor spot affixed to the center (Figure 2B). The main channel of the miniaturized flow chamber has the capacity to hold a volume of 12 μL (Figure2C), whereas the previous chambers of the HOBIC model had a volume of approximately 250 µL. This corresponds to a reduction in volume by a factor of 20.

In order to facilitate the optical readout of the sensor spot via an optical fiber, the flow chamber body was designed with a recess situated beneath the main channel (Figure 2D). The recess also permits sufficient space for polishing the underside of the 3D printing material for enhanced transparency. A sealing ring was pressed between two 3D-printed retainers in order to hold the optical fiber in a position directly underneath the sensor spot. The top of the flow chamber consisted of a 3D-printed lid with a glued-in glass pane which was pressed onto the main body by screws to allow the chamber to be opened for later analyses (Figure 2D). Initially, a more flexible polycarbonate foil was inserted into the lid and tested. However, the foil bulged while the glass pane was more stable under flow. A sealing ring was inserted into a cut-out in the main body in order to prevent leakages without bending the thin lid. The upper part of the flow chamber was designed to be as thin as possible in order to facilitate the scanning of the entire main chamber with microscopes during subsequent analyses. Additional sealing rings were placed around the inlet and outlet to prevent leakage between the 3D-printed threads and Luer adapters. Several designs with integrated 3D-printed Luer connectors were tested but discarded, due to leaks occurring after heat steam sterilization. The material costs for a fully assembled chamber amount to less than 20 $, and the vast majority of components (all except for the lid and the sensor spots) are reusable. Compared to the previous chambers, which had a fabrication cost of 400 $ each and were fully reusable following disinfection and thorough cleaning, this amounts to an expense reduction of factor 20.

### 3.2 Surface characterization of 3D printing material

**FIGURE 3:**
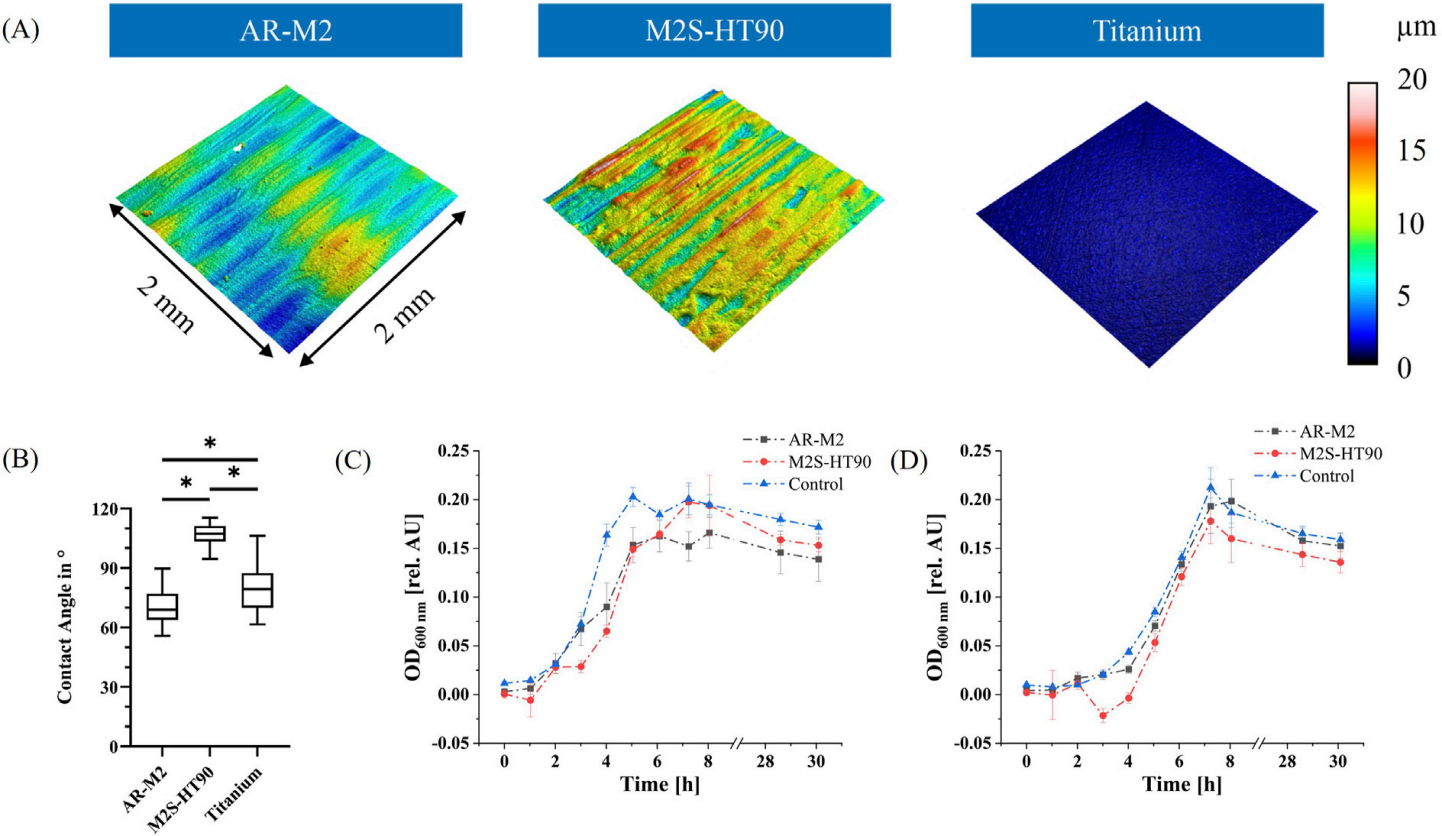
Property analysis of 3D printing materials. **(A)**: Representative images of surface topography of the 3D printing materials and titanium control. Colors show different heights in the respective profile. **(B)**: Static water contact angle measurements on the surfaces of the two 3D printing materials and titanium control disks. Statistically significant differences between materials with *p* ≤ 0.05 are depicted by (*). **(C)**: Bacterial biocompatibility evaluation of the two 3D printing materials AR-M2 (black) and M2S-HT90 (red) towards *S. oralis*. Statistically significant differences between materials for each time point with *p* ≤ 0.05 are given in Supplementary Table S2. **(D)**: Bacterial biocompatibility evaluation of the two 3D printing materials with a mixture of five oral bacteria: *S. oralis*, *A. naeslundii*, *V. dispar*, *F. nucleatum* and *P. gingivalis*. Statistically significant differences between materials for each time point with *p* ≤ 0.05 are given in Supplementary Table S3. BHI medium served as a control to show unhindered growth in **(C, D)**.

The surface topography of 3D-printed disks produced by the two printers and materials – as well as the titanium control – were analysed via confocal microscopy. As shown in Figure 3A, the surface topography of the two 3D printing materials was different. The values of three different roughness parameters are shown in Table 1. The mean value of all roughness parameters for M2S-HT90 was found to be twice as high as for AR-M2. Compared to the titanium sample, both 3D-printed samples are 10 to 20-fold rougher. Within each roughness parameter, all differences between materials were statistically significant.

**TABLE 1:** Surface topography parameters Rz, Ra and Rmax for the two 3D printing materials AR-M2 and M2S-HT90 as well as titanium, as measured by confocal microscopy.

Material	Rz [µm]	Ra [µm]	Rmax [µm]
AR-M2	3.35 ± 0.36	0.79 ± 0.08	3.65 ± 0.39
M2S-HT90	6.81 ± 0.79	1.26 ± 0.25	7.71 ± 0.96
Titanium	0.32 ± 0.09	0.03 ± 0.01	0.38 ± 0.17

Additionally, static water contact angle measurements were conducted (Figure 3B) to characterize the surface of the 3D printing materials and titanium control disks. The water contact angle of the 3D printing material M2S-HT90 was approximately 107 (± 5.5)°, while AR-M2 and titanium exhibited contact angles of approximately 70 (± 8.5)° and 80 (± 11.3)°, respectively. For all three comparisons between materials, differences were statistically significant.

### 3.3 Bacterial biocompatibility of 3D printing material

In order to ascertain the potentially inhibitory effect of leachables from the two 3D printing materials on the growth of bacteria that are used in the biofilm model, biocompatibility tests following ISO 10993-12 were performed. Extraction medium was prepared from both 3D printing materials. A visible increase in turbidity of the medium was observed during the extraction period for both printing materials. The growth of *S. oralis*, as well as of the mixture of the five bacteria used in the biofilm model, was then monitored in the extraction medium by measuring the OD_600_ (see Figures 3C, D). The growth of *S. oralis* and of a mixture of the five bacteria in regular BHI medium served as controls. After a short lag phase, exponential growth was observed for all samples, before transitioning into the stationary/dead phase. At the time point of the highest optical density of the control during the cultivation of *S. oralis*, optical densities were observed to be approximately 25% lower for both printing materials compared to controls. Mixed-effects analysis confirmed that the differences between the 3D printing materials and the control were statistically significant (adjusted *P* value <0.0001), while the difference between the two materials themselves was not significant (adjusted *P* value 0.81). For the mixture of the five bacterial species, the materials AR-M2 and M2S-HT90 showed 9% and 16% lower values in optical density after 7 h, respectively. The mixed-effects analysis revealed that only the difference between the M2S-HT90 material and the control was statistically significant (adjusted *P* value 0.02).

### 3.4 Bacterial growth and pH development in the microfluidic flow chamber system

**FIGURE 4:**
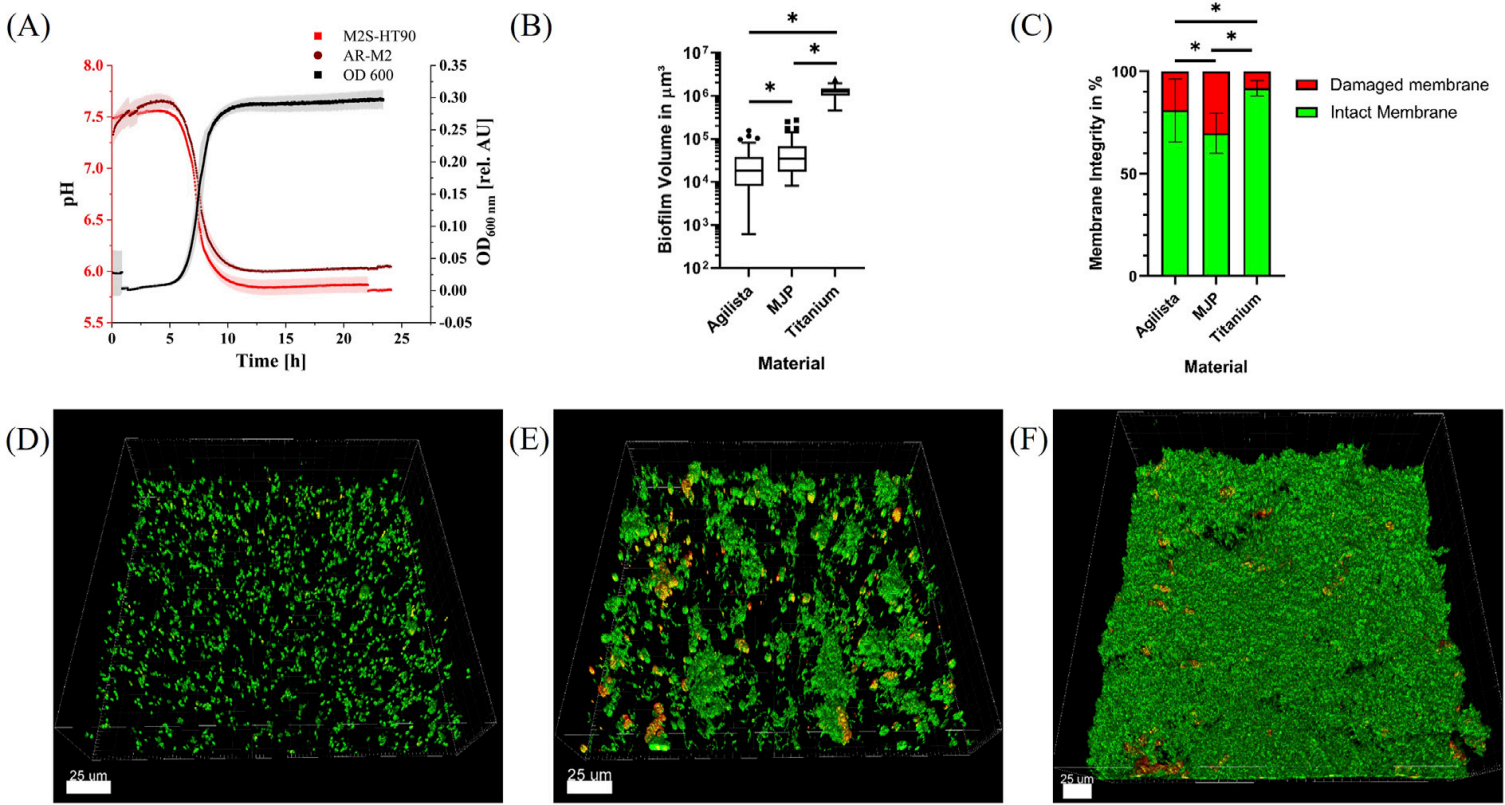
Biofilm growth in 3D-printed microfluidic flow chambers. **(A)**: OD_600_ of the five bacterial species inside the culture bottle during biofilm experiments for 24 h (black) and pH value inside the 3D-printed flow chambers (light red and dark red). **(B)**: Volume of biofilms per 185 μm × 185 μm grown on indicated specimen. Statistically significant differences with *p* ≤ 0.05 are indicated by (*). **(C)**: Membrane integrity of bacteria inside the biofilms grown on indicated specimen. Statistically significant differences with *p* ≤ 0.05 are indicated by (*). **(D**–**F)**: Representative CLSM images of 185 μm × 185 μm sections of live/dead-stained five-species biofilms grown for 24 h in flow chambers fabricated using the 3D printing materials AR-M2 **(D)** and M2S-HT90 **(E)** and on titanium specimen in the flow chambers described by Kommerein et al. (2018) **(F)**. Bacteria with intact membrane are depicted in green, whereas bacteria with damaged membrane are shown in red/orange.

*S. oralis*, *A. naeslundii*, *V. dispar*, *F. nucleatum* and *P. gingivalis* were grown anaerobically inside a culture bottle and introduced into the flow chamber system containing 3D-printed flow chambers from both materials via a peristaltic pump. The bacterial growth within the culture bottle was monitored by inline measurements of the OD_600_. The growth curve is depicted in Figure 4A. After a lag phase of approximately 5 h, exponential growth was observed (black curve). This growth ultimately reached an OD_600_ of 0.28, followed by the bacteria progression to the stationary phase after roughly 10 h. In addition, the pH value was measured via a sensor spot inside the main channel of the flow chamber as also shown in Figure 4A (red curves). The pH value decreased from 7.5 to a steady-state value of 6 and 5.8 after approximately 12 h for the 3D printing materials AR-M2 and M2S-HT90, respectively. With a *P* value of 0.0451, this difference was statistically significant.

### 3.5 Biofilm growth in the microfluidic flow chamber system

Biofilms were grown anaerobically in the 3D-printed flow chambers at 37°C for 24 h, stained with the fluorescent dyes Syto9 and propidium iodide (PI), and subsequently analyzed via confocal scanning laser microscopy (CSLM). Some of the flow chambers could not be analyzed due to a broken glass cover or air bubbles within the main channel which resulted in n = 11 and n = 12 biofilm samples for live/dead staining of the AR-M2 and M2S-HT90 3D printing material, respectively. Z-stack images of the samples were reconstructed into three-dimensional biofilms (Figures 4D–F) for which the biofilm volume (Figure 4B) as well as the live/dead distribution (Figure 4C) were calculated. Biofilms grown on the 3D printing material AR-M2 showed a mean volume of 2.9 × 10^4^ ± 3.1 × 10^4^ μm^3^ whereby a proportion of ∼86% exhibited an intact membrane. Strong fluorescent background signals were observed after staining of the biofilms, resulting in some variations of biofilm volume and live/dead distributions. In flow chambers made of M2S-HT90 printing material, a proportion of ∼71% of biofilms grown were found to have an intact membrane whilst their average volume was 5.3 × 10^4^ ± 5.5 × 10^4^ μm^3^. In comparison to the 3D printing materials, biofilms grown on titanium disks (n = 9) showed a volume and intact membrane proportion of 1.2 × 10^6^ ± 3.8 × 10^5^ μm^3^ and ∼92%, respectively. The differences in biofilm volume and the proportion of damaged or intact membranes grown on the respective materials were statistically significant.

### 3.6 Qualitative species distribution of biofilms grown in 3D-printed microfluidic flow chambers

**FIGURE 5:**
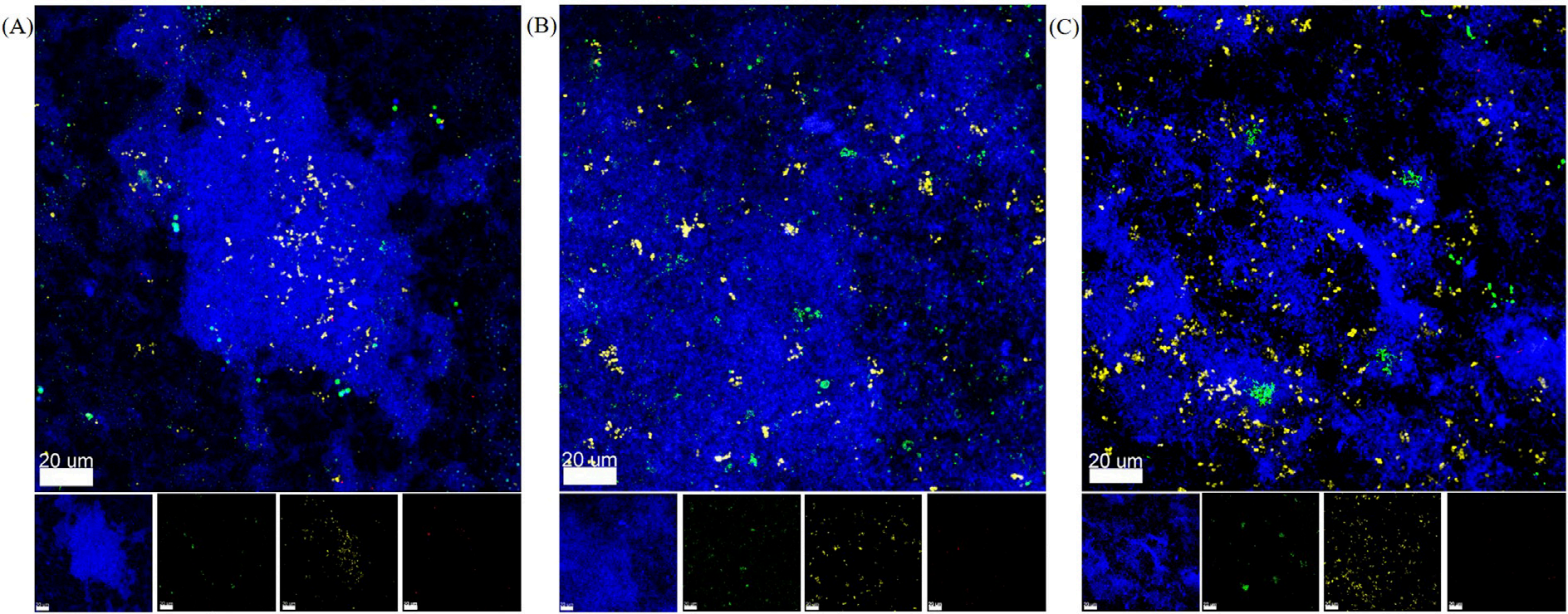
Representative FISH-staining of biofilm species distribution after 24 h in flow chamber system. Overlay and separate images of the four channels analyzed by CLSM with *S. oralis* in blue, *A. naeslundii* in green, *V. dispar* in yellow, *P. gingivalis* in red, and *F. nucleatum* in red and blue. **(A)** Biofilm in microfluidic flow chamber made of AR-M2 material. **(B)** Biofilm in microfluidic flow chamber made of M2S-HT90 material. **(C)** Biofilm on titanium specimens in the HOBIC model.

To qualitatively confirm the presence of all five bacterial species within the biofilms, species-specific FISH staining was performed and analyzed by CLSM. Exemplary images for each material are shown in Figures 5A–C. *S. oralis* is stained in blue, *A. naeslundii* in green, *V. dispar* in yellow, *F. nucleatum* in co-localized blue and red, and *P. gingivalis* in red. *F. nucleatum* and *P. gingivalis* can also be distinguished from each other based on their shape – with *F. nucleatum* showing up as long, thin strings and *P. gingivalis* as small dots or rods. In all chambers, the main component of the biofilm was observed to be *S. oralis*. Less abundant (but spread out throughout the biofilms and with similar amounts), *A. naeslundii* and *V. dispar* colonies were observable. For *P. gingivalis*, only a small number of single bacteria were visible in some places of the biofilms. *F. nucleatum* could not be seen in the sampled spots in the 3D-printed chambers. On titanium, a single *F. nucleatum* bacterium was found.

## 4 Discussion

Peri-implantitis poses a major challenge in the field of modern dentistry. The complexity of oral bacterial biofilms necessitates *in vitro* models that allow for the analysis of biofilm characteristics in a controlled environment. In this work, a 3D-printed microfluidic and optically accessible flow chamber was developed to grow an oral five-species biofilm directly on the 3D-printed surface. A pH-sensitive sensor spot was integrated into the main channel of the flow chamber to monitor the pH value in close proximity to the growing biofilms. The biofilm growth was compared in flow chambers fabricated with two different acrylate-based 3D printing materials, as well as in the established model system on titanium disks.

By miniaturizing the flow chambers of the HOBIC model, the amounts of materials used in each experiment can be decreased considerably. Especially due to the large amount of nutrient medium required while operating the flow chamber system under constant flow and the high costs of fluorescent dyes, this amounts to a substantial reduction of necessary expenses. Furthermore, the use of 3D printing technology allows for the fabrication of cost-effective flow chambers that are easily adaptable. To demonstrate such a modification, a pH-sensitive sensor spot was integrated into the chamber for effortless and constant monitoring of pH directly at the biofilm interface, which was not possible with previous HOBIC model flow chambers. In order to allow a direct comparison between the 3D-printed microfluidic flow chambers presented in this work and the HOBIC model, it was necessary to match the flow velocities of the two systems. For this, the flow velocities in the center of the main channel of the initial model were calculated, and the flow rate of 100 μL/min (which was described as representing saliva flow in hibernation mode allowing for increased biofilm formation) was reduced to 25 μL/min in the 3D-printed chambers. This reduced the medium consumption by 75% and also reduced the amount of fluorescent dyes necessary for analysis. However, any further size reduction of the system should be considered with caution, as the rapid biofilm growth can quickly clog channels thus limiting the possible cultivation time.

In order to test whether toxic substances might leach out of the 3D-printed parts and hinder bacterial growth, bacterial biocompatibility tests were performed for both 3D printing materials according to ISO 10993-12. 3D-printed cubes were incubated in cell culture medium to obtain extraction medium. Subsequently, bacteria were cultivated in the extraction medium, and growth curves were obtained by measuring the OD_600_. While 3D Systems declare their printing material to be biocompatible in the polymerized form, it is strongly recommended to confirm this for each individual application (3D Systems, 2022). For example, Siller et al. (2020) demonstrated the biocompatibility of the 3D printing material AR-M2 for adipose tissue-derived mesenchymal stem/stroma cells, whereas Grab et al. (2021) observed the opposite effect with respect to human endothelial cells. For the printing material M2S-HT90, similar studies were conducted by Winkler et al. (2022) – who found the material to be biocompatible for mouse fibroblast cells and *Saccharomyces cerevisiae*, but not for human embryonic kidney cells. Since different organisms and media were used in this work, the biocompatibility of both printing materials was specifically examined with respect to our application. *S. oralis* has been described as an early colonizer of oral bacterial biofilms, thereby forming the initial biofilm to which other bacteria can adhere (Kolenbrander et al., 2010; Wake et al., 2016). It was shown that within the HOBIC model, *S. oralis* was the primary contributor to early stage biofilms (Kommerein et al., 2018). Accordingly, *S. oralis* was also tested alone in our biocompatibility experiments. The presence of both printing materials was found to hinder the growth of the five bacterial species, with the greatest impact observed for *S. oralis*. This suggests that although the tested bacteria do grow in the presence of the respective 3D printing materials, initial biofilms will form more slowly on the 3D-printed surfaces – probably being due to unpolymerized monomers leaching out of the 3D printing material over time. The greater resilience of the bacteria within the mixture towards the effects of the extraction medium may be due to supporting inter-species interactions. To enhance the biocompatibility of the 3D printing materials, additional and prolonged incubation periods in ethanol could be implemented to extract potentially toxic substances from the printed components. A negative effect on bacterial growth by the titanium samples was not expected, as this material has approved highly biocompatible properties (Sidambe, 2014), which was also specifically confirmed by the high viability of biofilms grown on titanium specimens in the HOBIC model (Kommerein et al., 2018).

It has been reported that several surface characteristics – including but perhaps not limited to charge, roughness, and hydrophobicity – can influence biofilm formation (Song et al., 2015). While bacteria can adhere to hydrophobic (θ > 90°) as well as hydrophilic surfaces (θ < 90°) (Busscher et al., 2010; Law, 2014), superhydrophobic and superhydrophilic surfaces have been shown to reduce bacterial attachment (Zhang et al., 2013; Zheng et al., 2021). Although one of the materials was slightly hydrophobic and the other was slightly hydrophilic, the water contact angles measured for the 3D printing materials and titanium in this study do not fall within the aforementioned extreme categories, and therefore no negative effect on the biofilm growth was anticipated. A rougher surface is expected to increase the contact area between cells and the surface and therefore benefit bacterial attachment (Teughels et al., 2006; Setter et al., 2023). In comparison to the titanium control and the 3D printing material AR-M2, the M2S-HT90 printing material exhibited the highest surface roughness, thus indicating comparatively more favorable conditions for biofilm growth.

During biofilm experiments in the flow system, the bacterial growth within the culture bottle was monitored via OD_600_ measurements using an inline photometer. The results showed a reproducible characteristic bacterial growth curve with lag, exponential, and stationary phases. The pH value inside the main channel of the flow chamber matched the course of the growth curve. In M2S-HT90 chambers, the pH was slightly lower than in the AR-M2 chambers. This could be attributed to the increased biofilm growth on M2S-HT90. As bacterial growth increases, pH values decline due to the production of acidic substances. In this *in vitro* model, the early colonizers *S. oralis* and *A. naeslundii* were expected to form the initial biofilms on the sample surfaces (Nyvad and Kilian, 1987) – and as part of its fermentative metabolism, *S. oralis* produces lactic acid, thereby lowering the pH value considerably (Soet et al., 2000). During dysbiosis of oral biofilms leading to peri-implant diseases, the species distribution is expected to shift towards gram-negative bacterial species, such as *P. gingivalis* (Hajishengallis, 2015). Furthermore, the metabolic activity of the early colonizers is expected to be downregulated due to limited nutrient supply in deeper layers of the biofilm, therefore resulting in less acidification (Radaic and Kapila, 2021). This also supports the growth of *P. gingivalis*, as low pH hinders its growth (Takahashi and Schachtele, 1990). For experiments involving longer-term biofilm growth (especially after replacing the culture bottle with fresh and sterile medium after initial biofilm development), a rise in pH is to be expected and could also be a marker for a dysbiotic shift. Given the limited duration of biofilm growth in this study, however, this shift was not anticipated.

Biofilms were grown for 24 h in the respective flow chambers before being live/dead stained and analyzed via CLSM. Image stacks were reconstructed to three-dimensional biofilms, and the biofilm volume and live/dead distribution based on membrane integrity were calculated. For all materials tested in this study, biofilm growth could be observed. The largest biofilm volume and most viable biofilm was observed on titanium specimens. This can be explained by the highly biocompatible properties of titanium. When calculated for volume per area, our biofilms on titanium showed three times higher values than the four-species biofilms reported by Kommerein et al. (2018). This may be attributed to the presence of *F. nucleatum* in the biofilms of the present study, which could support the growth of the entire biofilm. *F. nucleatum* acts as a bridging bacterium, co-aggregating with many oral species and thus adding structural stability to the biofilm and possibly increasing thickness (Bradshaw et al., 1998). The live proportion of approximately 90% reported by Kommerein et al. (2018) is consistent with our findings. In comparison to titanium, biofilms grown in the 3D-printed flow chambers showed both lower volume and less viability. We hypothesize that in addition to the lower biocompatibility of both 3D printing materials described above, significantly lower biofilm volume and viability may be due to the 3D-printed surface properties, which could be less favorable for bacterial attachment. This could either be due to chemical or physical surface properties, or some combination thereof. The chemical compositions of the printing materials are not known, but the higher surface roughness of the M2S-HT90 printing material may be the reason for the thicker biofilms observed in comparison to the AR-M2 material. Furthermore, the latter showed substantial background signals following fluorescent staining – resulting in challenges while setting the threshold during image analysis and, thus, leading to larger variations in the respective parameters. Due to the imaging background noise, the chambers of both printing materials had to be inverted in order to achieve biofilm growth on the glass cover to allow for FISH analysis. Biofilm thickness on the glass appeared to be similar to thickness on the printing materials, although the details of the two biofilm properties diverged. The qualitative results on species composition showed four of the five species introduced into the system in the final biofilms. *F. nucleatum* was the only species that could not be detected in the 3D-printed chambers. Given that only a limited number of flow chambers were subjected to qualitative FISH analysis, it is possible that *F. nucleatum* simply contributed to the biofilm in a too low amount to be detected. This would be in line with another study conducted in parallel that quantified species distribution in HOBIC biofilms on titanium by qRT-PCR, which showed that less than 0.1% of biofilm bacteria consisted of *F. nucleatum* (data not shown). Additionally, due to the similar coloration to *P. gingivalis*, these cells may have also been misidentified or overlooked. For example, if they were present in an unfavorable orientation in the biofilm, it could have been difficult to distinguish these bacterial species based on their morphologies.

In conclusion, in this study we successfully developed a low-cost, 3D-printed microfluidic flow chamber that proved to be suitable for growing reproducible multispecies oral biofilms *in vitro* while simultaneously maintaining low medium consumption. The transparent 3D printing materials allowed for the optical readout of parameters of interest (as demonstrated for the pH value) in close proximity to the growing biofilms. Furthermore, this system was compatible with analyses that require microscopic readout without opening the chamber, thus, preserving the biofilm’s native morphology. If necessary, opening and accessing the biofilm for further analyses was also possible. Due to the advantages of additive manufacturing technology, the flow chamber design used within this system could be readily adapted to include further optical sensors or titanium disks to more closely replicate the *in vivo* peri-implant situation. Although slower biofilm growth was observed on the 3D-printed surfaces, nonetheless these 3D-printed microfluidic chambers supported growth and maturation of the biofilms over 24 h. Overall, this system provides a promising platform for future high-throughput *in vitro* investigations of implant-associated infections.

## Data Availability

The raw data supporting the conclusions of this article will be made available by the authors, without undue reservation.

## References

[B1] 3D Systems (2022). VisiJet M2S-HT90 datasheet. Available at: https://www.3dsystems.com/materials/visijet-m2s-ht90-mjp (Accessed June 13, 2024).

[B2] Al-Ahmad A. Muzafferiy F. Anderson A. C. Wölber J. P. Ratka-Krüger P. Fretwurst T. (2018). Shift of microbial composition of peri-implantitis-associated oral biofilm as revealed by 16S rRNA gene cloning. J. Med. Microbiol. 67, 332–340. 10.1099/jmm.0.000682 29458668

[B3] Apatzidou D. Lappin D. F. Hamilton G. Papadopoulos C. A. Konstantinidis A. Riggio M. P. (2017). Microbiome of peri-implantitis affected and healthy dental sites in patients with a history of chronic periodontitis. Archives Oral Biol. 83, 145–152. 10.1016/j.archoralbio.2017.07.007 28780383

[B4] Benoit M. R. Conant C. G. Ionescu-Zanetti C. Schwartz M. Matin A. (2010). New device for high-throughput viability screening of flow biofilms. Appl. Environ. Microbiol. 76, 4136–4142. 10.1128/aem.03065-09 20435763 PMC2897429

[B5] Blanc V. Isabal S. Sanchez M. C. Llama‐Palacios A. Herrera D. Sanz M. (2014). Characterization and application of a flow system for *in vitro* multispecies oral biofilm formation. J. periodontal Res. 49, 323–332. 10.1111/jre.12110 23815431

[B6] Bradshaw D. J. Marsh P. D. Watson G. K. Allison C. (1998). Role of Fusobacterium nucleatum and coaggregation in anaerobe survival in planktonic and biofilm oral microbial communities during aeration. Infect. Immun. 66, 4729–4732. 10.1128/iai.66.10.4729-4732.1998 9746571 PMC108582

[B7] Busscher H. J. Rinastiti M. Siswomihardjo W. van der Mei H. C. (2010). Biofilm formation on dental restorative and implant materials. J. Dent. Res. 89, 657–665. 10.1177/0022034510368644 20448246

[B8] Ceri H. Olson M. E. Stremick C. Read R. R. Morck D. Buret A. (1999). The Calgary Biofilm Device: new technology for rapid determination of antibiotic susceptibilities of bacterial biofilms. J. Clin. Microbiol. 37, 1771–1776. 10.1128/jcm.37.6.1771-1776.1999 10325322 PMC84946

[B9] Corbin A. Pitts B. Parker A. Stewart P. S. (2011). Antimicrobial penetration and efficacy in an *in vitro* oral biofilm model. Antimicrob. agents Chemother. 55, 3338–3344. 10.1128/aac.00206-11 21537022 PMC3122404

[B10] Costerton J. W. Lewandowski Z. Caldwell D. E. Korber D. R. Lappin-Scott H. M. (1995). Microbial biofilms. Annu. Rev. Microbiol. 49, 711–745. 10.1146/annurev.mi.49.100195.003431 8561477

[B11] Davies D. (2003). Understanding biofilm resistance to antibacterial agents. Nat. Rev. Drug Discov. 2, 114–122. 10.1038/nrd1008 12563302

[B12] Díez-Aguilar M. Morosini M. I. Köksal E. Oliver A. Ekkelenkamp M. Cantón R. (2018). Use of Calgary and microfluidic BioFlux systems to test the activity of fosfomycin and tobramycin alone and in combination against cystic fibrosis Pseudomonas aeruginosa biofilms. Antimicrob. agents Chemother. 62, 016500-17–e11128. 10.1128/aac.01650-17 PMC5740371 29084746

[B13] Donlan R. M. Costerton J. W. (2002). Biofilms: survival mechanisms of clinically relevant microorganisms. Clin. Microbiol. Rev. 15, 167–193. 10.1128/cmr.15.2.167-193.2002 11932229 PMC118068

[B14] Dreyer H. Grischke J. Tiede C. Eberhard J. Schweitzer A. Toikkanen S. E. (2018). Epidemiology and risk factors of peri‐implantitis: a systematic review. J. periodontal Res. 53, 657–681. 10.1111/jre.12562 29882313

[B15] Flemming H.-C. Wingender J. (2010). The biofilm matrix. Nat. Rev. Microbiol. 8, 623–633. 10.1038/nrmicro2415 20676145

[B16] Fu J. Zhang Y. Lin S. Zhang W. Shu G. Lin J. (2021). Strategies for interfering with bacterial early stage biofilms. Front. Microbiol. 12, 675843. 10.3389/fmicb.2021.675843 34168632 PMC8217469

[B17] Ghezzi D. Boi M. Sassoni E. Valle F. Giusto E. Boanini E. (2023). Customized biofilm device for antibiofilm and antibacterial screening of newly developed nanostructured silver and zinc coatings. J. Biol. Eng. 17, 18. 10.1186/s13036-023-00326-y 36879323 PMC9987098

[B18] Grab M. Stieglmeier F. Emrich J. Grefen L. Leone A. König F. (2021). Customized 3D printed bioreactors for decellularization—high efficiency and quality on a budget. Artif. Organs 45, 1477–1490. 10.1111/aor.14034 34219220

[B19] Gross B. C. Erkal J. L. Lockwood S. Y. Chen C. Spence D. M. (2014). Evaluation of 3D printing and its potential impact on biotechnology and the chemical sciences. ACS Publ. 86, 3240–3253. 10.1021/ac403397r 24432804

[B20] Hajishengallis G. (2015). Periodontitis: from microbial immune subversion to systemic inflammation. Nat. Rev. Immunol. 15, 30–44. 10.1038/nri3785 25534621 PMC4276050

[B21] Heitz Mayfield L. J. A. Salvi G. E. (2018). Peri‐implant mucositis. J. Clin. periodontology 45, S237–S245. 10.1111/jcpe.12953 29926488

[B22] Henkel AG & Co.KGaA (2020). Technical data sheet Loctite® EA M-31CL. Available at: https://datasheets.tdx.henkel.com/LOCTITE-EA-M-31CL-en_GL.pdf (Accessed May 30, 2024).

[B23] Høiby N. Bjarnsholt T. Moser C. Bassi G. L. Coenye T. Donelli G. (2015). ESCMID guideline for the diagnosis and treatment of biofilm infections 2014. Clin. Microbiol. Infect. 21, S1–S25. 10.1016/j.cmi.2014.10.024 25596784

[B24] International Organization for Standardization (2021). Biological evaluation of medical devices. ISO 10993-12. Venier, Switzerland: ISO copyright office.

[B25] Jepsen S. Berglundh T. Genco R. Aass A. M. Demirel K. Derks J. (2015). Primary prevention of peri‐implantitis: managing peri‐implant mucositis. J. Clin. periodontology 42, S152–S157. 10.1111/jcpe.12369 25626479

[B26] Kolenbrander P. E. Palmer R. J. Periasamy S. Jakubovics N. S. (2010). Oral multispecies biofilm development and the key role of cell–cell distance. Nat. Rev. Microbiol. 8, 471–480. 10.1038/nrmicro2381 20514044

[B27] Kommerein N. Doll K. Stumpp N. S. Stiesch M. (2018). Development and characterization of an oral multispecies biofilm implant flow chamber model. PLoS One 13, e0196967. 10.1371/journal.pone.0196967 29771975 PMC5957423

[B28] Koyanagi T. Sakamoto M. Takeuchi Y. Ohkuma M. Izumi Y. (2010). Analysis of microbiota associated with peri-implantitis using 16S rRNA gene clone library. J. Oral Microbiol. 2, 5104. 10.3402/jom.v2i0.5104 PMC3084566 21523229

[B29] Kristensen M. F. Leonhardt D. Neland M. L. B. Schlafer S. (2020). A 3D printed microfluidic flow-cell for microscopy analysis of *in situ*-grown biofilms. J. Microbiol. methods 171, 105876. 10.1016/j.mimet.2020.105876 32087186

[B30] Law K.-Y. (2014). Definitions for hydrophilicity, hydrophobicity, and superhydrophobicity: getting the basics right. J. Phys. Chem. Lett. 5, 686–688. 10.1021/jz402762h 26270837

[B31] McGlennen M. Dieser M. Foreman C. M. Warnat S. (2023). Using electrochemical impedance spectroscopy to study biofilm growth in a 3D-printed flow cell system. Biosens. Bioelectron. X 14, 100326. 10.1016/j.biosx.2023.100326

[B32] Nance W. C. Dowd S. E. Samarian D. Chludzinski J. Delli J. Battista J. (2013). A high-throughput microfluidic dental plaque biofilm system to visualize and quantify the effect of antimicrobials. J. Antimicrob. Chemother. 68, 2550–2560. 10.1093/jac/dkt211 23800904 PMC3797639

[B33] Nyvad B. Kilian M. (1987). Microbiology of the early colonization of human enamel and root surfaces *in vivo* . Scand. J. Dent. Res. 95, 369–380. 10.1111/j.1600-0722.1987.tb01627.x 3477852

[B34] Purevdorj B. Costerton J. W. Stoodley P. (2002). Influence of hydrodynamics and cell signaling on the structure and behavior of Pseudomonas aeruginosa biofilms. Appl. Environ. Microbiol. 68, 4457–4464. 10.1128/aem.68.9.4457-4464.2002 12200300 PMC124093

[B35] Radaic A. Kapila Y. L. (2021). The oralome and its dysbiosis: new insights into oral microbiome-host interactions. Comput. Struct. Biotechnol. J. 19, 1335–1360. 10.1016/j.csbj.2021.02.010 33777334 PMC7960681

[B36] Rath H. Stumpp S. N. Stiesch M. (2017). Development of a flow chamber system for the reproducible *in vitro* analysis of biofilm formation on implant materials. PLoS One 12, e0172095. 10.1371/journal.pone.0172095 28187188 PMC5302373

[B37] Recupido F. Toscano G. Tatè R. Petala M. Caserta S. Karapantsios T. D. (2020). The role of flow in bacterial biofilm morphology and wetting properties. Colloids Surfaces B Biointerfaces 192, 111047. 10.1016/j.colsurfb.2020.111047 32388030

[B38] Roberts F. A. Darveau R. P. (2002). Beneficial bacteria of the periodontium. Periodontology 2000, 30. 10.1034/j.1600-0757.2002.03004.x 12236894

[B39] Sanz Martin I. Doolittle‐Hall J. Teles R. P. Patel M. Belibasakis G. N. Hämmerle C. H. F. (2017). Exploring the microbiome of healthy and diseased peri‐implant sites using Illumina sequencing. J. Clin. periodontology 44, 1274–1284. 10.1111/jcpe.12788 PMC7209775 28766745

[B40] Setter O. P. Jiang X. Segal E. (2023). Rising to the surface: Capturing and detecting bacteria by rationally-designed surfaces. Curr. Opin. Biotechnol. 83, 102969. 10.1016/j.copbio.2023.102969 37494819

[B41] Sidambe A. T. (2014). Biocompatibility of advanced manufactured titanium implants—a review. Materials 7, 8168–8188. 10.3390/ma7128168 28788296 PMC5456424

[B42] Siller I. G. Enders A. Gellermann P. Winkler S. Lavrentieva A. Scheper T. (2020). Characterization of a customized 3D-printed cell culture system using clear, translucent acrylate that enables optical online monitoring. Biomed. Mater. 15, 055007. 10.1088/1748-605x/ab8e97 32348964

[B43] Smeets R. Henningsen A. Jung O. Heiland M. Hammächer C. Stein J. M. (2014). Definition, etiology, prevention and treatment of peri-implantitis–a review. Head & face Med. 10, 34–13. 10.1186/1746-160x-10-34 25185675 PMC4164121

[B44] Soares G. M. S. Teles F. Starr J. R. Feres M. Patel M. Martin L. (2015). Effects of azithromycin, metronidazole, amoxicillin, and metronidazole plus amoxicillin on an *in vitro* polymicrobial subgingival biofilm model. Antimicrob. agents Chemother. 59, 2791–2798. 10.1128/aac.04974-14 25733510 PMC4394767

[B45] Soet J. J. Nyvad B. Kilian M. (2000). Strain–related acid production by oral Streptococci. Caries Res. 34, 486–490. 10.1159/000016628 11093023

[B46] Song F. Koo H. Ren D. (2015). Effects of material properties on bacterial adhesion and biofilm formation. J. Dent. Res. 94, 1027–1034. 10.1177/0022034515587690 26001706

[B47] Takahashi N. Schachtele C. F. (1990). Effect of pH on the growth and proteolytic activity of Porphyromonas gingivalis and Bacteroides intermedius. J. Dent. Res. 69, 1266–1269. 10.1177/00220345900690060801 2191980

[B48] Teughels W. van Assche N. Sliepen I. Quirynen M. (2006). Effect of material characteristics and/or surface topography on biofilm development. Clin. oral implants Res. 17, 68–81. 10.1111/j.1600-0501.2006.01353.x 16968383

[B49] Tsagkari E. Connelly S. Liu Z. McBride A. Sloan W. T. (2022). The role of shear dynamics in biofilm formation. NPJ biofilms microbiomes 8, 33. 10.1038/s41522-022-00300-4 35487949 PMC9055050

[B50] Wake N. Asahi Y. Noiri Y. Hayashi M. Motooka D. Nakamura S. (2016). Temporal dynamics of bacterial microbiota in the human oral cavity determined using an *in situ* model of dental biofilms. NPJ biofilms microbiomes 2, 16018–16019. 10.1038/npjbiofilms.2016.18 28721251 PMC5515266

[B51] Wang G. P. (2015). Defining functional signatures of dysbiosis in periodontitis progression. Genome Med. 7, 40. 10.1186/s13073-015-0165-z 25926890 PMC4414443

[B52] Winkler S. Meyer K. V. Heuer C. Kortmann C. Dehne M. Bahnemann J. (2022). *In vitro* biocompatibility evaluation of a heat‐resistant 3D printing material for use in customized cell culture devices. Eng. Life Sci. 22, 699–708. 10.1002/elsc.202100104 36348657 PMC9635007

[B53] Zhang X. Wang L. Levänen E. (2013). Superhydrophobic surfaces for the reduction of bacterial adhesion. Rsc Adv. 3, 12003–12020. 10.1039/c3ra40497h

[B54] Zhang Y. Li Y. Yang Y. Wang Y. Cao X. Jin Y. (2022). Periodontal and peri-implant microbiome dysbiosis is associated with alterations in the microbial community structure and local stability. Front. Microbiol. 12, 785191. 10.3389/fmicb.2021.785191 35145492 PMC8821947

[B55] Zheng S. Bawazir M. Dhall A. Kim H.-E. He L. Heo J. (2021). Implication of surface properties, bacterial motility, and hydrodynamic conditions on bacterial surface sensing and their initial adhesion. Front. Bioeng. Biotechnol. 9, 643722. 10.3389/fbioe.2021.643722 33644027 PMC7907602

[B56] Zitzmann N. U. Berglundh T. (2008). Definition and prevalence of peri‐implant diseases. J. Clin. periodontology 35, 286–291. 10.1111/j.1600-051x.2008.01274.x 18724856

