# Supplementary material for: Optically accessible, 3D-printed flow chamber with integrated sensors for the monitoring of oral multispecies biofilm growth in vitro
Source: Front Bioeng Biotechnol. 2024 Nov 11;12:1483200. doi: 10.3389/fbioe.2024.1483200 (PMC11586212; doi:10.3389/fbioe.2024.1483200)
Supplement: Supplementary file 1 [file Table1.DOCX]

Supplementary Material

# Supplementary Data

Table SI1: FISH probes.

| So405 | ACA gCC TTT AAC TTC AgA CTT ATC TAA | Alexa Fluor 405 |
| --- | --- | --- |
| An488 | Cgg TTA TCC AgA AgA Agg gg | Alexa Fluor 488 |
| Vd568 | AAT CCC CTC CTT CAg TgA | Alexa Fluor 568 |
| Pg647 | CAA TAC TCg TAT CgC CCg TTA TTC | Alexa Fluor 647 |
| FUS664-blau | CTT gTA gTT CCg CYT ACC TC | Alexa Fluor 405 |
| FUS664-rot | CTT gTA gTT CCg CYT ACC TC | Alexa Fluor 647 |

*Table SI2: Statistically significant differences between materials for each time point with p ≤ 0.05 for figure 2A.*

| Time point (hours) | AR-M2 vs. M2S-HT90 | AR-M2 vs. Titanium | M2S-HT90 vs. Titanium |
| --- | --- | --- | --- |
| 0 |  | * | * |
| 1 |  | * | * |
| 2 |  |  |  |
| 3 | * |  | * |
| 4 |  | * | * |
| 5 |  | * | * |
| 6 |  | * | * |
| 7 | * | * |  |
| 8 |  | * |  |
| 28 |  | * | * |
| 29 |  | * | * |

*Table SI3: Statistically significant differences between materials for each time point with p ≤ 0.05 for figure 2B.*

| Time point (hours) | AR-M2 vs. M2S-HT90 | AR-M2 vs. Titanium | M2S-HT90 vs. Titanium |
| --- | --- | --- | --- |
| 0 |  | * | * |
| 1 |  |  |  |
| 2 |  | * |  |
| 3 | * |  | * |
| 4 | * | * | * |
| 5 | * | * | * |
| 6 | * |  | * |
| 7 |  |  | * |
| 8 | * |  |  |
| 28 |  |  | * |
| 29 | * |  | * |
